# Factors Affecting Non-Adherence among Patients Diagnosed with Unipolar Depression in a Psychiatric Department of a Tertiary Hospital in Kolkata, India

**DOI:** 10.1155/2013/809542

**Published:** 2013-12-04

**Authors:** Sohini Banerjee, Ravi Prasad Varma

**Affiliations:** Sree Chitra Tirunal Institute for Medical Sciences and Technology, Thiruvananthapuram, Kerala 695011, India

## Abstract

Non-adherence to depression treatment is a common clinical problem globally. However, limited research is available from India. This cross-sectional study aimed to assess non-adherence to prescribed treatment among patients with unipolar depression at a psychiatric out-patient department (OPD) of a tertiary hospital in Kolkata, India. The Morisky Medication Adherence Scale (MMAS) was used and a questionnaire designed by the Principal Investigator (PI) was administered. A total of 239 patients with unipolar depression were interviewed of whom 66.9% (160) were non-adherent and 33.1% (79) were adherent to treatment. The difference was significant (Fisher's Exact <0.000). Women were nearly three times at a higher risk of being non-adherent compared to men (OR 2.7; 95% CI 1.0–7.1). The non-adherent group compared to the adherent group was significantly more likely to consume extra medicines than the recommended amount (OR 2.8; 95% CI 1.1–7.3) and had lower internal locus of control (LOC) (OR 4.5; 95% CI 2.4–8.3). Adherence to prescribed treatment in an out-patient clinical setting was a problem among patients with unipolar depression. Suitable interventions on individuals with the above mentioned attributes are required in India and in similar settings where non-adherence to depression therapy is an important public health problem.

## 1. Introduction

Adherence (“extent to which a person's behaviour corresponds with medical or health advice provided by a health care provider”) [1, 2] to therapy is emerging as a major public health challenge globally—both for communicable (tuberculosis, HIV/AIDS) and noncommunicable (depression, diabetes) diseases. The consequences of poor/non-adherence are extensive. It negatively impacts treatment effectiveness thus resulting in poor therapeutic outcomes. Non-adherence in some instances could result in serious complications requiring the individual to be hospitalised. This not only adds considerable physical strain and mental agony to the individual and the family but results in economic burden as well. It also adds pressure on the health system. Moreover, research indicates medication non-adherence may have a damaging effect on the individual's health related quality of life (QOL) [3].

Non-adherence to treatment is a well-documented issue in the care of unipolar or major depression. According to World Health Report, 1999 [4] and the Global Burden of Disease (GBD) Update, 2004 [5] in 1990, depression was the fourth leading cause of overall disease burden and is emerging as a major public health challenge with regard to its prevalence, morbidity, mortality (suicide) and financial ramifications [5]. Various determinants such as nature and duration of therapy, disease characteristics, medication side-effects, cost of treatment, characteristics of health service facilities, the relation between the physician and patient, patient characteristics such as socioeconomic factors, patient's perspective about the illness and therapy have been reported to influence adherence.

The World Health Organisation (WHO) categorised the determinants of non-adherence into five dimensions: social and economic, health system-related, therapy-related, condition-related, and patient-related [3]. Reported rates of non-adherence to prescribed medication in the management of unipolar depression vary considerably. Evidence suggests that more than 30.0% to 60.0% of those diagnosed with depression terminate their therapy prematurely without physician's approval [6, 7]. From a review of 32 studies on adherence to depression therapy, Pampallona et al. reported medication compliance rates of 14 epidemiological studies which ranged from 30.0% to 97.0% (rates reported may be affected by the small sample size) [8].

A meta-analysis of studies conducted between 1975 and 1996 in the USA showed that patients on antidepressants took an average of more than three-fifths (65.0%) of the prescribed amount as compared to 76.0% adherence in physical disorders [9]. Research by DiMatteo and colleagues, 2000 indicate that those diagnosed with unipolar depression are three times more likely to be noncompliant regarding their prescribed medical advice in general in comparison to nondepressed patients [10]. A study by Patel and colleagues documented the increasing disease burden of unipolar depressive disorders in India [11]. The study from Goa, India highlighted patient-reported reasons for non-adherence, but the findings were limited by the small sample size (*n* = 36). “Not finding time for treatment due to work” was the most commonly cited reason of non-adherence (50.0%). Improvement in the condition and caring for a family member were reported by 19% of the respondents. Other reasons cited included distance from residence to health centre, lengthy waiting time in the hospital, adverse effect of medication [12]. The study from Chandigarh, India on the other hand conducted a study among 50 individuals diagnosed with mild and moderate unipolar depression and only explored patient's attitudes and beliefs towards antidepressant medications and their adherence to treatment. This study underscored the importance of patients' beliefs about antidepressants which influence adherence to medication. Findings of this study indicated that the longer the individual was in treatment for depression adherence decreased. The small sample size restricts the generalisability of the study findings. The study provided a partial insight into the issue of adherence by focusing only on patient's beliefs about antidepressants. It did not examine other correlates that affect adherence such as health system related and treatment related factors [13].

Few studies from India have aimed to comprehensively assess factors affecting non-adherence to prescribed treatment among those diagnosed with unipolar depression. This motivated the current study which aimed to assess correlates (sociodemographic variables, therapy associated factors, impact of multi-drug treatment and co-morbidities, locus of control, reasons) of non-adherence among patients diagnosed with unipolar depression in a psychiatric department of a tertiary hospital in Kolkata, India.

## 2. Methodology

### 2.1. The Setting

This cross-sectional study was conducted among adult patients visiting the psychiatric out-patient department (OPD) of a tertiary hospital in Kolkata. This is one of the oldest medical colleges and tertiary care public hospitals well connected by all modes of transportation-buses, trains, trams, and the underground metro. Easy accessibility makes it one of the busiest hospitals in the city. The psychiatric OPD in the hospital is open on six days a week. Patients pay a nominal fee of Rs 2.00 for a card registering them for consultation. Prescribed medicines are written on this OPD card and can be used on multiple occasions. Medications such as amitriptyline, fluoxetine, sertaline (antidepressants), and clonazepam (anxiolytic) among others are dispensed free of cost to the patient from the hospital dispensary. The psychiatric department (including indoor facilities) and the OPD are housed in two separate buildings. The clinical team comprises 10 senior psychiatrists including the head of department, professors and medical officers. Other staffs include interns, post-graduate trainees, nurses, security staff and a documentation officer. Although there is no provision for counsellors or psychiatric social workers in the department, there is one social worker for the entire hospital. In the absence of psychiatric social workers and the busy schedule of the doctors, by rotation the nursing staffs assist in the OPD by explaining to patients how prescribed medicines have to be taken. To aid the process of medication intake, medicines are dispensed in small white paper packets (separate ones for different medicines) on which nurses write instructions in Bengali. Bengali is the common language spoken and understood by all—both patients and medical professionals.

### 2.2. Sample Size

The sample size planned for this study was 305 which arrived based on information provided by a study conducted by Chakraborty and colleagues in 2009 [13]. As per this study, the expected proportion of patients who missed more than 25.0% of the doses over a three month period was 12.0%. Setting the worst acceptable level at 8.0% and confidence interval at 95.0%, a sample size of 254 was concluded using EPI Info 6 (Stat Calc). After 20.0% correction for dropouts/non-compliance was made, the final sample size was calculated to be 305.

### 2.3. Sample Selection Procedures

Adult patients, both men and women attending the psychiatric OPD and had been diagnosed with unipolar depression at least six months prior to the commencement of the study, were approached. The upper age limit of 60 years was selected to rule out the possibility of including patients with dementia. Only those willing to participate were recruited in the study. All physicians were made aware of the study and they agreed to send patients who met the inclusion criteria. In spite of best efforts, some patients may have been missed from this overcrowded OPD but, this number was negligible. Moreover, the register containing patient details recorded the patient prescription number only (no diagnosis). The problem was further compounded by the fact that each individual was not provided with a unique identification number whereby tracing patients missed from the OPD register could not be considered as an option for inviting them to participate in the research.

#### 2.3.1. Inclusion Criteria

- Patients meeting ICD-10 [14] criteria of unipolar depression as diagnosed by the consultant psychiatrist and referred.
- Men and women between 18–60 years of age.
- In treatment for at least six months prior to commencement of study.
- Currently in remission—diagnosed as “unipolar depression in remission” (the definition of remission offered by Zimmerman and colleagues), 2006 was followed: symptomatic remission judged by the clinician and functional recovery (e.g., presence of features of positive mental health such as optimism and self-confidence; a return to one's usual, normal self; and a return to usual level of functioning) as judged by the patient [15].
- Other co-morbid conditions excluding patients with psychotic features, diagnosed bipolar affective disorder.
- Willing to participate and provide written informed consent.

#### 2.3.2. Exclusion Criteria

- Information exclusively from care givers.
- Individuals refusing to participate.

### 2.4. Data Collection

Data collection was carried out by the principal investigator (PI) only after obtaining informed/understood consent. The 8-item Morisky Medication Adherence Scale (MMAS), a validated instrument, was administered to measure adherence. The MMAS is a reliable tool that has been widely used to measure adherence particularly among hypertensive patients. It was developed in 2008 and is a modified version of the 4-item MMAS. The MMAS is an 8-item structured instrument where seven questions have dichotomous (Yes, No) responses. There are five response options in the eighth question. It has a reliability of 0.83 along with good predictive validity. Although the application of this tool is limited in the domain of mental health, nevertheless it is an appropriate instrument for measuring specific medication behaviour in chronic diseases [16].

The cross-language equivalence procedure was followed to adapt the Bengali version of MMAS. The tool was translated in Bengali and back translated into English by two independent persons, one a social scientist and the other a school teacher, none of whom were part of the research. Their agreement was 98.6%. Face validation of MMAS was carried out by two psychiatrists, one from Kolkata and the other from United Kingdom (UK), none of whom were involved with the study and their agreement was 99.1%.

In addition, a semi-structured interview schedule was developed by the PI to explore demographic and treatment-related factors, the impact of multi-drug treatment, co-morbidities, and LOC on adherence. The interview schedule was designed after considerable literature review and consultations with social scientists, epidemiologists, psychiatrists, and psychologists at Sree Chitra Tirunal Institute for Medical Sciences and Technology (SCTIMST), Achutha Menon Centre for Health Sciences Studies (AMCHSS), and a consultant psychiatrist from UK. The interview schedule was translated into Bengali by two independent individuals, one a social scientist and the other a school teacher, none of whom were involved in the research endeavour. The interview schedule was pretested in the OPD which took no more than 30 minutes. Modifications and suggestions were incorporated in the final version of the interview schedule. The interview schedule was divided into four sections, namely, demography (Section 1), treatment and adherence related (Section 2), multi-drug treatment and co-morbidities (Section 3), and locus of control (LOC: Section 4). In addition, notes were maintained to record various observations. Prior to conclusion of the interview patients were asked to comment on methods of improving adherence to prescribed medication. Interview was finished by thanking the respondent for their time and forbearance.

*Duration of Data Collection*. Data were collected from mid-June to mid-September 2011 (21.06.11–14.09.11) at the OPD, six days a week, Monday to Friday from 9:30 a.m. to 2:00 p.m. and Saturday 9:30 a.m. to 12:00 noon (OPD timings).

### 2.5. Ethical Considerations

Ethical clearance was obtained from both Technical Advisory Committee (TAC), Institutional Ethics Committee (IEC) of SCTIMST and the collaborating institute. Permission to use the MMAS for the present study was requested and granted. Participants were provided written and verbal communication about the purpose of the study, contacts of persons concerned, respondent's right to discontinue the interview at any time they so deemed without affecting their treatment benefits.

### 2.6. Data Entry

Data were entered by the PI in SPSS (Statistical Package for Social Sciences) version 17.0 for windows (SPSS, Inc., Chicago, IL). Data were cleaned and random checks were done for missing values and inconsistencies with the hard copy. Hard copies of the questionnaire and signed consent forms are in safe and secure custody of the PI.

### 2.7. Data Analysis

Data analysis was also carried out by the PI using SPSS 17.0 for windows (SPSS, Inc., Chicago, IL). Univariate analysis of all variables was carried out to describe the sample characteristics. Further analysis has been carried out with non-adherence as the outcome of interest. Bivariate analyses of independent variables with dependent/outcome variable were done by cross-tabulation and testing with Pearson Chi square. A multivariate model was fitted using Binary Logistic Regression (enter method) with non-adherence as outcome variables. Variables found significant in the bivariate analysis and considered to have an interaction effect were included. This was done to adjust for possible confounding and interactions to arrive at model explaining the dependent variable. Measure of association was presented as Odds Ratio (OR) with 95.0% Confidence Interval (CI). Sex disaggregated analysis was attempted. Multivariate analysis was attempted with high and moderate (0) and poor adherence (1) as the outcome variable.

## 3. Results

A total of 246 participants were approached of whom 239 signed the consent form and 7 declined. Thus, the response rate was calculated as 239/246 = 97.2% and the coverage achieved was 239/305 = 78.4%.

### 3.1. Sociodemographic Characteristics of Sample

Among the total of 239 subjects, nearly three quarters (176; 73.6%) were women. One-third of the study participants were in the age group of 31–40 years (71, 29.8%). Majority of the participants were Muslims (137; 57.3%), and the remaining 102 (42.7%) were Hindus. The study subjects were predominantly married (173; 72.4%), and majority of them had some education (124; 51.9%). More than half (128; 53.6%) of the respondents were residing in rural areas. The most frequently reported occupation was being a housewife or performing household chores (145; 60.7%). More than three quarters of the respondents reported belonging to nuclear families (185; 77.4%). The median age of the sample was 40.0 years ± 11.0 (range 18–60); median number of family members was 4 ± 2.7 (range 1–16), and the median average of monthly income was Indian Rupees (INR *₹*) 4, 000 ± 4230.4 (range 500–25,000) (Table 1).

### 3.2. Treatment and Non-Adherence

**Figure 1:**
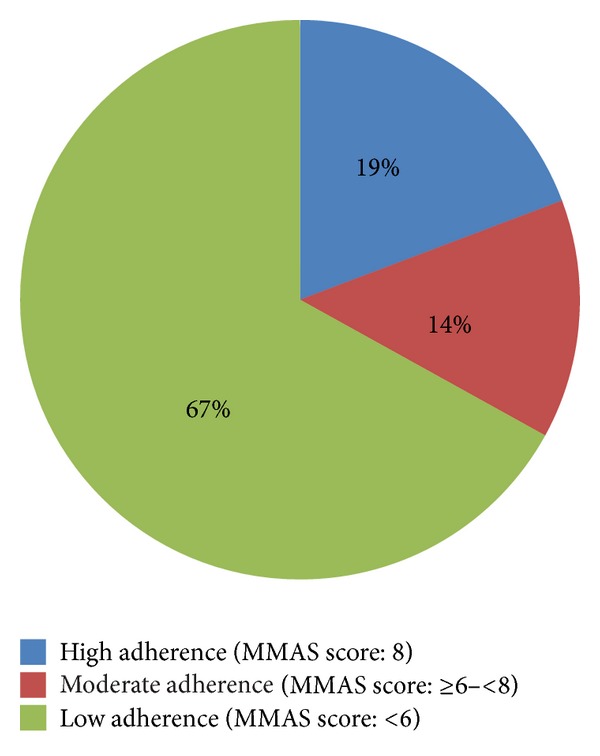
Hospital-based prevalence of adherence (*N* = 239).

Based on the scoring of the MMAS, majority of the patients were found to have poor adherence (160; 66.9%). A few (33; 13.8%) were moderately adherent while 46 (19.2%) were highly adherent. The median score was 4.75 ± 2.42 (Figure 1).

An overwhelming majority (214; 89.5%) had no idea about the diagnosis of the condition for which they were seeking treatment. A mere 23 (9.6%) had some idea about their condition of which only 2 (0.9%) used the term “depression.” The average duration (in months) of treatment was 18 ± 16.1.

### 3.3. Medication

The mean number of medications advised was 3.3 ± 1.0. All patients were prescribed antidepressants of whom majority were prescribed the TCA (Tricyclic antidepressants) and tetracyclic classes (133; 55.6%) of antidepressants. In addition, Benzodiazepines were the most frequently (224; 93.7%) prescribed drugs (Table 2).

### 3.4. Recommended Dose of Prescribed Medication

Most of the study participants were recommended to take medicines thrice a day (108; 45.2%) followed by twice a day (Table 3).

### 3.5. Missed Follow-Up Treatment/Visit

A total of 163 (68.2%) respondents confirmed having missed follow-up treatment while 76 (31.8%) stated they were able to be present at the hospital as per their scheduled visit. Of the 163 who were unable to be present on days of their consultation few reported having missed their follow up treatment usually (27; 16.6%) (Table 4).

### 3.6. Multi-Therapy Treatment and Patient Satisfaction

Of the 239 patients interviewed most of the patients reported using multiple therapy. Nearly three quarters (174; 72.8%) of the patients reported having taken more medicines than prescribed. A few reported visiting healing temples (73; 30.5%), healers of other medical system. One of the reasons they offered for seeking extra medical help was dissatisfaction with the current management of their condition (Table 5).

### 3.7. Co-Morbidities

A total of 43 (18.0%) individuals reported having another health condition for which medical treatment was being sought—34 (17.3%) reported having one, 7 (3.6%) reported two and 2 (1.0%) reported three physical conditions respectively (Table 6). The commonly reported co-morbidities included hypertension and thyroid related illnesses. All 43 individuals were prescribed medicines for their co-morbidities of whom more than three-fourths reported that consumption of medicines for their co-morbid conditions did not make it difficult for them to adhere to their treatment for unipolar depression (Table 7).

### 3.8. Locus of Control and Health Outcomes

Half the respondents (120; 50.2%) stated that they were responsible for their own health and 110 (46.0%) maintained that remaining healthy depended on one's own self. However, as less as one thirds of the respondents (72, 30.1%) believed in having command of their own health or recovery in case of illness. Beliefs about bad luck and karma as causes of illness were voiced by many. A lesser number of people were of the opinion that God's will or evil eye were responsible for the illness (76; 31.8%). While majority of the participants held on to the notion that cure depended on luck/fate a lesser number believed in God's contribution in the recovery process (Table 8).

### 3.9. Advice and Adherence

Not only medication but ability to follow advice is also considered an important component of adherence to prescribed treatment and hence participant's views about the issue were explored. Of the 239 respondents only 23 (9.6%) reported that they were given some advice apart from prescribed medicines for example, blood test, particularly for ruling out thyroid related illnesses (8; 34.8%), counselling (3; 13.4%), life style modifications (morning walks, healthy diet—3; 13.4%), and others (9; 39.1%). Of these 23 individuals approximately half (11; 47.8%) reported inability to follow suggested counsel while the remaining 12 (52.2%) expressed that they had no problem in doing so. Financial constraint was the most frequently reported reason for not being able to follow advice while lack of time and space were reported as reasons for not being able to change lifestyle.

For the purpose of further analysis adherence was converted into a binary variable which based on the MMAS scores were classified as adherent (0; high and moderate adherence) and non-adherent (1; low adherence). Based on this classification, 79 (33.1%) were found to be adherent and more than three-fifths (160; 66.9%) were found to be non-adherent.

In addition, to the MMAS when patients were asked if they missed medication in the last thirty days, 145 (60.7%) gave positive response while 94 (39.3%) replied negatively.

### 3.10. Factors Associated with Non-Adherence

Bivariate analysis of outcome variable was done with other independent variables. Adherence scores were used to categorise respondents into a binary variable adherent and non-adherent. The association was considered significant if “*P*” value was less than 0.05. Bivariate analysis of all variables was done. However, only four independent variables were found to be significantly associated with poor adherence which is listed here. The odds of women being non-adherent as compared to men were 2.4 times. Compared to those who were adherent there was an 18.5 odds that the non-adherent ones would stop medication in the last 30 days as compared to their adherent counterpart. Similarly, there were significant associations between taking more medication and LOC (Table 9).

*Multivariate Analysis*. The Multivariate Binary Logistic Regression Model was fitted using the enter method. The covariates found significant in the bivariate analysis were included as were those which were not significant, yet considered important in influencing the outcome-adherence. Women were found to be nearly three times at a higher risk of being non-adherent compared to men (OR 2.7; CI 1.0–7.1). The non-adherent group compared to the adherent group was significantly more likely to consume extra medicines than the recommended number and had a considerably lower internal LOC (Table 10).

### 3.11. Patient Reported Barriers to Adherence

Since medication non-adherence is a common feature of any chronic disease, information regarding factors hindering adherence is crucial in designing interventions promoting adherence to treatment for those suffering from chronic diseases. Considering this aspect a set of additional questions were asked to 193 patients who reported experiencing difficulty in complying with the prescribed treatment. Distinction was made in classifying barriers to adherence which were patient related and those which were not. The following section documents some of the most frequently reported reasons for missing medication.  Table 11 describes the patient-reported barriers to adherence. Forgetting to take prescribed medicines (109; 56.5%) was the most commonly reported cause for missing medication among the 193 who expressed inconvenience in following treatment regimen. Distance between home and health care facility from which treatment was being sought was cited as another obstacle which prevented patients from remaining adherent to their prescribed treatment. Difficulty in taking medicines at the scheduled hour and the burden of household duties featured among the top ten reasons cited by those diagnosed with unipolar depression. Long waiting hours in the hospital (55; 28.5%), lack of clear explanations provided by health workers about medication intake (26; 13.5%), and dissatisfaction with the amount of time spent by the consulting physician in examining patients (15; 7.8%) and shortage of drug supply were some of the health system related reasons mentioned by the subsample reporting difficulty in maintaining prescribed treatment. Inability to visit hospital during working hours due to loss of wages was reported by nearly a quarter of those missing medication. Cost of medicines and paucity of funds for visiting the hospital were the finance related issues referred to by patients. Lack of social support in terms of reminder about medication intake, escorting patient to the hospital featured among the reasons for therapy discontinuation.

Although, it was not an explicit objective of the current study, the sample nevertheless warranted a sex-disaggregated analysis which is reported in the current section. As reported earlier there were 63 men and 176 women in the study.

There were an equal proportion of men and women in all age categories. While more than the half the men were Hindus (38, 60.3%), Muslim women accounted for 63.6% of the study population More than seventy percent of both men and women were married. A larger proportion of men were single (never married) compared to women while the number of widows outnumbered their male counterparts. The number of women with no education was nearly twice as men in the same category. More than half then men and women had some education (1 to 10 years). However, men were four times more likely to have higher education (greater than 10 years) compared to women. Only 5 (2.8%) of women reported being unemployed compared to 16 (25.4%) men. Being a housewife or involvement with household activities was reported as the most common occupation by the women (140; 79.5%) while the most commonly mentioned occupation among men was irregular employment (18; 28.6%). Exactly twice as many men were involved in regular employment as compared to women. There was nearly equal proportion of men and women from rural areas as were from the peri-urban and urban areas. More than three-fourths of both men and women belonged to nuclear families (Table 1).

These observations suggest that gender differentials in adherence are very important but generally unaddressed currently. There is a need for gender-sensitive research in future studies.

## 4. Discussion

This study has made one of the earliest attempts to document factors adversely affecting adherence to treatment among those diagnosed with unipolar depression in a psychiatric department of a tertiary hospital in Kolkata. Unipolar depression is one of the most common mental disorders: point prevalence is 1.9% for men and 3.2% for women [1]. The emergence of depression as a chronic disease is well documented in the current literature [11, 17–19]. Although inability to adhere to prescribed treatment is a ubiquitous phenomenon among those with chronic conditions [7, 10] however, epidemiological data were lacking.

Based on the findings of available literature, the aim of this work was to explore the problem of adherence associated with depression, a chronic disease. This research findings are notable with respect to six issues: the hospital based prevalence of non-adherence, significant risk of women being non-adherent, internal locus of control, medication intake, no knowledge about the diagnosis of the condition for which treatment is being sought and inclusion of a single question to address adherence.

### 4.1. Hospital Based Prevalence of Non-Adherence

The hospital based prevalence of non-adherence to antidepressants in this study was found to be 66.9%. This is considerably higher than the reported rates of non-adherence to depression medication which range from 10.0% to 60.0% (median 40.0%) [7, 8, 20]. Other descriptive epidemiological studies have validated that one out of three persons are unable to complete therapy [8]. One probable explanation for this high rate of non-adherence could be the class of medication prescribed. More than half the participants in the study were being prescribed TCAs and TeCA as compared to those treated with SSRIs. There is conflicting evidence about the efficacy of SSRIs over other groups of antidepressants, particularly TCAs. While some studies have documented higher rates of treatment discontinuation in patients advised antidepressants other than SSRIs [21, 22]. Other meta-analyses reported no influence of the class of drugs being prescribed on drop-out rates [23]. Further research in this area is warranted. While the type of drugs prescribed may have a bearing on maintenance of therapy nevertheless, it provides a partial insight into the complex and nuanced phenomenon of non-adherence. Other factors known to impact non-adherence include characteristics of the disease, disease therapies, patient associated aspects including beliefs, social and economic support. High non-adherence indicates the need to improve adherence among those undergoing treatment for depression. Additional clinical training of medical personnel, initiate awareness among patients and caregivers about depression and its associated problem of therapy discontinuation, addressing accessibility issues, and ensuring availability of commonly prescribed are some appropriate measures that may support adherence and non-adherence.

### 4.2. Women More Prone to Being Non-Adherent

In this study, women were found to be more non-adherent compared to men (OR 2.7; 95% CI 1.1–7.1). Available literature on this issue is inconsistent. Similar to this study finding, studies from Canada reported better compliance to treatment among men [24]. However, findings contrary to this study have been reported from USA and Belgium where men were more likely to discontinue treatment without physician consent [25, 26]. This divergence in reported findings may be due to socio-cultural differences. The multiple roles assumed by woman include that of home makers, professionals, spouse, mothers, and care providers may contribute to their inability to adhere to prescribed regimen. More women were likely to report that they found it difficult to visit the hospital as they had to attend to household activities while more men reported they lost wages or were not permitted leave from work. Inability to visit health facilities during working days may have contributed in keeping men away from health facilities; the reason for the study population being predominantly women. This however remains speculative and merits further research. Since more women are reported to suffer from depression and seek treatment, educating them about the potential problems related to abrupt suspension of therapy is crucial. Education about this widespread yet disregarded clinical phenomenon will help to prevent relapses and ensure better outcomes.

### 4.3. Low Internal Locus of Control among the Non-Adherents

This study noted that adherence was moderated by locus of control. Consistent with existing literature a low locus of control was noted among the non-adherent in this study. The relationship between LOC and adherence in other health conditions, particularly diabetes is well documented [27]. However, there is a dearth of epidemiological data establishing this association between LOC and depression. A low internal LOC indicates an individual's belief that the main causes of events in their lives are governed by events beyond their own personal control. Reinforcing a belief that individuals have command over their own lives, especially with reference to health outcomes is vital in improving adherence. Physicians can play an important role here. But, with demanding work schedules, they are already stretched beyond their capacities in this and similar other settings with low doctor to patient ratio and have little additional time to devote to patients. Recruiting psychiatric social workers, counsellors (which is lacking in the study setting) who would interact with patients addressing the issue of adherence and LOC may be an alternative and viable option.

### 4.4. Lack of Awareness about Diagnosis

An overwhelming majority of the study participants were not aware of the diagnosis of the health condition for which they were seeking health care. When probed, patients reported knowing the symptoms for which they were being treated but not the exact diagnosis (except in two instances). The possible reasons for this finding may be physician apathy to explain the diagnosis to the patients or patient inability to comprehend explanations offered. As mentioned above engaging additional personnel may resolve this problem. It is not uncommon for those afflicted by a stigmatising illness to refer to the symptoms rather than the diagnosis to make their illness more socially acceptable. In Asian cultures the explanatory models of mental health experiences highlight the associations of depression with common complaints such as excessive vaginal discharge [28]. A study on tuberculosis in Philippines by Nichter draws attention to the importance of illness semantics in the realm of health research. Tuberculosis is often referred to as “weak lungs” a terminology considered less prejudiced. He underscores the connection between illness perception and deleterious treatment practices. To ensure appropriate treatment practices among those with any form of stigmatising conditions, careful attention must be given to patient perception of the illness [29].

### 4.5. Inappropriate Intake of Medication

Non-adherent patients (160; 66.9%) were more likely to take more medicines than the prescribed dose than their adherent counterparts. While most studies on adherence to antidepressants focus on discontinuation of medication as a feature of non-adherence, a few studies have nevertheless focused on the issue of additional intake of prescribed medicines [13, 30]. Both, abrupt discontinuation of medicines and additional intake of medications have harmful effects. In order for patients to derive optimum benefit from recommended therapy, the importance of taking medicines as recommended needs to be impressed upon them. This study employed a simple probe (Have you ever taken more tablets than recommended to become better quickly?) to elicit information from patients on overuse of medication which can be applied in similar other settings.

### 4.6. Simple Probe to Assess Non-Adherence

Although the variable adherence was made operational using MMAS, an additional probe was used as a supportive variable—this turned out to have a strong independent association, suggesting that a simple question like “Have you ever missed or stopped medication in the last thirty days” would be able to elicit the same kind of information and may be found to be more appropriate in settings lacking adequate health personnel.

## 5. Limitations and Strengths

Despite limitations imposed by the cross-sectional nature of the survey, clinical setting, retrospective study design and patient reported adherence, the study offered a strong ethical advantage as the patient was not exposed to an experience different from that of routine care provision. Mental health continues to remain a neglected public health concern. From this perspective this study makes some important contributions. The use of a semi-structured interview schedule allowed for documenting both quantitative and qualitative data. Data collection was carried out by a single investigator thereby eliminating the possibility of interobserver bias. This epidemiological study makes the first attempt using the 8 item MMAS for measuring adherence to prescribed depression therapy in India.

## 6. Conclusion

This study has enumerated health-related issues that are typically neglected but which routinely confront health services. Non-adherence to prescribed therapy in a clinical out-patient setting was found to be a common problem among those diagnosed with unipolar depression, which was characterised by discontinuation of medicines without consulting the treating physician. Measures have been suggested to increase adherence and improve management of depression. This study has highlighted that non-adherent patients are likely not to follow the drug regimen prescribed to them. This finding emphasises the need to sensitise the patients about the importance or correct and proper drug intake. Additional resources in terms of personnel should be allocated to enhance communication with patients encouraging them to be adherent.

This study also indicates the need for intersectoral programmes linking health departments with other departments such as public works department (improvements in transportation and communication system) to ameliorate the problem of non-adherence of depression therapy. Mental health policies addressing the specific gender needs will contribute to increasing adherence. For instance, modifications in working days and hours of health facilities may increase presentation of cases with depression in the hospitals and increase adherence to treatment. Preliminary findings of this study merit further research, and intersectoral systems oriented approach to improve adherence is needed for effective strategies in India and settings elsewhere where non-adherence to depression therapy is an important public health problem.

## Figures and Tables

**Table 1:** Sociodemographic characteristics of the respondents (*N* = 239).

Sociodemographic characteristics	*N* = 239	%	Men	%	Women	%
Sex						
Men	63	26.4				
Women	176	73.6				
Age (years)						
18–30	55	23.0	14	22.2	41	23.3
31–40	71	29.8	16	25.4	55	31.2
41–50	67	28.0	20	31.7	47	26.7
51–60	46	19.2	13	20.7	33	18.8
Religion						
Hindus	102	42.7	38	60.3	64	36.4
Muslims	137	57.3	25	39.7	112	63.6
Marital Status						
Single (Never married)	17	7.1	11	17.5	6	3.4
Married	173	72.4	47	74.5	126	71.6
Widow/er	35	14.6	3	4.8	32	18.2
Separated	14	5.9	2	3.2	12	6.8
Education (in completed years)						
No education (0)	93	38.9	14	22.2	77	43.8
Some education (1–10)	124	51.9	36	57.2	90	51.1
Higher education (>10)	22	9.2	13	20.6	9	5.1
Occupation						
Unemployed	21	8.8	16	25.4	5	2.8
Housewife/household chores	145	60.7	5	7.9	140	79.5
Irregular employment	29	12.1	18	28.6	11	6.3
Regular employment	21	8.8	14	22.2	7	4.0
Agricultural work	11	4.6	9	14.3	2	1.1
Others (cooks, maids, etc.)	12	5.0	1	1.6	11	6.3
Residence						
Rural	128	53.6	31	49.2	97	55.1
Peri-urban	38	15.9	12	19.0	26	14.8
Urban	73	30.5	20	31.7	53	30.1
Family type						
Nuclear	185	77.4	49	77.8	136	77.3
Joint/extended	42	17.6	12	19.0	30	17.0
Others	12	5.0	2	3.2	10	5.7
Per capita income (INR;*₹*)						
Below 2500	74	30.9	1	1.6	1	0.6
2500–4000	69	28.9	19	30.2	53	30.1
Above 4000–6000	43	18.0	17	27.0	52	29.5
Above 6000	53	22.2	17	27.0	36	20.5

**Table 2:** Commonly prescribed medicines (*N* = 239).

Types of medicines	*N*	%
Antidepressants		
Selective serotonin reuptake inhibitors (SSRIs)	106	44.4
Others (Tricyclic, Tetracyclic)	133	55.6
Other medications		
Benzodiazepine	224	93.7
Others (Vitamins, antihypertensive, etc.)	15	6.3

**Table 3:** Recommended frequency of medication intake (*N* = 239).

Medication intake	*N*	%
Once a day	38	15.9
Twice a day	93	38.9
Thrice a day	108	45.2

**Table 4:** Patient reported frequency of consultations missed (*N* = 163).

Frequency of consultations missed	*N*	%
Rarely	72	44.2
Sometimes	64	39.2
Usually	27	16.6

**Table 5:** Multiple forms of treatment as reported by patients (*N* = 239).

Multiple forms of treatment	Yes	%	No	%
Visit healing temple	73	30.5	166	69.5
Take more pills	174	72.8	65	27.2
Seek medical advice from other physicians due to dissatisfaction with current treatment	230	96.2	9	3.8
Consult health-care providers practicing alternative medicine	19	7.9	220	92.1

**Table 6:** Patient reported co-morbidities (*N* = 239).

Co-morbidities	Yes	%	No	%
Any other health condition	43	18.0	196	82.0
Hypertension	22	9.2	217	90.8
Thyroid related illnesses	11	4.6	228	95.4
Diabetes	5	2.1	234	97.9
Cardiovascular diseases	5	2.1	234	97.9
Others (bronchial asthma, etc.)	8	3.3	231	96.7

**Table 7:** Difficulty in adherence due to treatment regimen of co-morbid conditions (*N* = 43).

Degree of difficulty	*N*	%
Not at all	33	76.7
Sometimes	6	14.0
Rarely	4	9.3

**Table 8:** Locus of control and health outcomes (*N* = 239).

Locus of control	Yes	%	No	%
Internal locus of control				
Directly responsible for own health	120	0.2	119	49.8
Remaining healthy depends on self	110	46.0	129	54.0
In control of own health	75	31.4	164	68.6
If sick, recovery depends on self	72	30.1	167	69.9
External locus of control				
Illness as a result of bad luck/ill fate	145	60.7	94	39.3
Illness as a result of karma	107	44.8	132	55.2
Illness as a result of God's will	76	31.8	163	68.2
Illness as a result of evil eye	43	18.0	196	82.0
Recovery depends upon luck/fate	143	59.8	96	40.2
Recovery depends upon God	109	45.6	130	54.4

**Table 9:** Factors associated with poor adherence: results of bivariate analysis.

Variables	Non-adherent		Adherent		Total	%	Odds ratio	95% CI
	*N* = 160	%	*N* = 79	%				
Sex**								
Men	33	52.4	30	47.6	63	26.4	1	
Women	127	72.2	49	27.8	176	73.6	2.4	1.4–4.3
Stopped medication (30 days)**								
No	30	31.9	64	68.1	94	39.3	1	
Yes	130	89.7	15	10.3	145	60.7	18.5	9.2–36.8
Take more medicines**								
No	110	63.2	64	36.8	174	72.8	1	
Yes	50	76.9	15	23.1	65	27.2	1.9	1.0–3.7
Internal LOC**								
Yes	25	41.0	36	59.0	61	25.5	1	
No	135	75.8	43	24.2	178	74.5	4.5	2.4–8.3

**P < 0.001.

**Table 10:** Factors associated with poor adherence: multiple logistic regression.

Variables	Adjusted odds ratio	95% CI	*P* value
Sex*			
Men	1		
Women	2.7	1.1–7.1	**0.045**
Age group			
18–30	1		
31–40	0.8	0.3–2.1	0.632
41–50	0.4	0.1–1.2	0.149
51–60	0.3	0.1–1.0	0.051
Education (years completed)			
0	1		
1–10	1.3	0.6–2.8	0.486
>10	0.2	0.1–0.6	0.131
Religion			
Hindus	1		
Muslims	1.1	0.4–2.9	0.917
Marital status			
Never married	1		
Married	0.4	0.1–1.9	0.241
Separated	2.8	0.2–41.6	0.562
Widow/er	0.3	0.1–2.3	0.243
Occupation			
Unemployed	1		
Housewife/household chores	0.4	0.8–2.1	0.446
Irregular employment	1.9	0.2–19.8	0.681
Regular employment	2.8	0.3–31.5	0.403
Agricultural work	0.1	0.1–0.8	0.031
Others (cooks, maids)	0.2	0.2–1.3	0.192
Family type			
Nuclear	1		
Joint/extended	0.8	0.2–3.1	0.842
Others	6.7	0.6–79.8	0.119
Take more medicines**			
No	1		
Yes	2.8	1.1–7.3	**0.033**
Visited healing temple			
No	1		
Yes	0.6	0.2–1.5	0.371
Average treatment duration (months)			
<12	1		
12–24	0.4	0.1–0.9	0.010
>24	0.6	0.2–1.6	0.467
External LOC			
Yes	1		
No	0.1	0.2–3.7	0.951
Internal LOC**			
Yes	1		
No	4.5	2.4–8.3	**0.000**

**P < 0.001; *P < 0.05.

**Table 11:** Patient reported barriers to adherence of prescribed treatment (*N* = 193).

Patient reported barriers to adherence	Yes	%	No	%
Patient related and social factors				
Forget to take medicines	109	56.5	84	43.5
Difficult to take medicines at specified time	72	37.3	121	62.7
Did not feel like taking medicines (medication fatigue)	63	32.6	130	67.4
Too much of work at home	55	28.5	138	71.5
Unable to visit hospital working hours due to loss of wages	44	22.8	149	77.2
Stop medication for religious reasons	43	22.3	150	77.7
Did not get leave from work	26	13.5	167	86.5
Medicines were costly to buy	24	12.4	169	87.6
No one at home to remind about taking medications	21	10.9	172	89.1
Did not want others to know	19	9.8	174	90.2
Suggested by someone within family to stop medication	19	9.8	174	90.2
No one to accompany me to the hospital	19	9.8	174	90.2
Did not feel current treatment is effective	16	8.2	177	91.8
Afraid of side effects	13	6.7	180	93.3
Personal other illness (gall bladder operation, asthma, etc.)	11	5.7	182	94.3
Do not believe medicines will improve condition	10	5.2	183	94.8
Someone outside the family suggested stopping medicines	9	4.7	184	95.3
Could not arrange money to come to the hospital	7	3.6	186	96.4
Medication related				
Afraid of medication dependency	53	27.5	140	72.5
Feel worse after taking medication	36	18.7	157	81.3
Lack of proper explanation on how to take medicines	26	13.5	167	86.5
Too many medicines to take	22	11.4	171	88.6
Health-facility related				
Hospital is far from home	92	47.7	101	52.3
Long waiting hours in the hospital	61	31.6	132	68.4
No direct transportation to the hospital; change many times	47	24.4	146	75.6
Doctor did not spend enough time to examine	15	7.8	178	92.2
Medicines provided by hospital not available at the facility	5	2.6	188	97.4
Miscellaneous				
Inclement weather particularly rains	19	9.8	174	90.2
Difficulty in swallowing medicines	6	3.1	187	96.9
Medical store did not have supply of medicines prescribed	5	2.6	188	97.4
